# Effect of M3 muscarinic acetylcholine receptor deficiency on collagen antibody-induced arthritis

**DOI:** 10.1186/s13075-016-0926-0

**Published:** 2016-01-19

**Authors:** Janet Beckmann, Nicole Dittmann, Iris Schütz, Jochen Klein, Katrin Susanne Lips

**Affiliations:** Laboratory of Experimental Trauma Surgery, Justus-Liebig University Giessen, Kerkraderstrasse 9, 35394 Giessen, Germany; Department of Pharmacology, School of Pharmacy, Goethe-University Frankfurt, Max-von-Laue Strasse 9, 60438 Frankfurt am Main, Germany

**Keywords:** Muscarinic acetylcholine receptor, Non-neuronal cholinergic system, Rheumatoid arthritis, Collagen antibody-induced arthritis, Joint destruction, Synovial inflammation

## Abstract

**Background:**

There is increasing evidence that the non-neuronal cholinergic system might be of importance for the pathology of rheumatoid arthritis. The role of M3 muscarinic acetylcholine receptor (M3R) in this regard has, however, not been investigated to date. Thus, in the present study we analyzed if M3R deficiency might have a protective effect on experimentally induced arthritis.

**Methods:**

Collagen antibody-induced arthritis (CAIA) was evoked in M3R-deficient (M3R^−/−^) mice and wild-type (WT) littermates. Severity of arthritis was assessed by scoring of paw swelling. The joints of arthritic and nonarthritic animals were analyzed for histopathological changes regarding synovial tissue, cartilage degradation and bone destruction. Further, gene expression analysis of respective markers was performed. Systemic and local inflammatory response was determined by flow cytometry and immunohistochemistry for leukocytes as well as mRNA and protein measurements for pro-inflammatory cytokines and chemokines.

**Results:**

In arthritic M3R^−/−^ mice the number of leukocytes, specifically neutrophils, was enhanced even though clinical arthritis score was not significantly different between WT and M3R^−/−^ mice with CAIA. In M3R^−/−^ mice, levels of neutrophil chemoattractant chemokine C-X-C-motif ligand 2 (CXCL2) as well as the pro-inflammatory cytokine interleukin-6 were already strongly increased in mice with low arthritis score, whereas WT mice only showed prominent expression of these markers when reaching high arthritis scores. Furthermore, arthritic M3R^−/−^ mice displayed a stronger degradation of collagen II in the articular cartilage and, most strikingly, histopathological evaluation revealed more severe bone destruction in arthritic mice with M3R deficiency compared to WT littermates. Moreover, in M3R^−/−^ mice, gene expression of markers for bone degradation (matrix metalloproteinase 13, cathepsin K and receptor activator of nuclear factor-κB ligand) was already increased in mice with low arthritis score.

**Conclusions:**

Taken together, the present study shows that while M3R^−/−^ mice were not protected from CAIA, they had a tendency toward a higher inflammatory response after arthritis induction than WT mice. Further, arthritis-induced joint destruction was significantly stronger in mice with M3R deficiency, indicating that stimulation of M3R might have protective effects on arthritis.

## Background

Rheumatoid arthritis (RA) is a chronic inflammatory autoimmune disease, characterized by synovial inflammation resulting in cartilage destruction and bone erosion [1]. Current therapies such as classical disease-modifying antirheumatic drugs (DMARDs) and biologicals (e.g., anti-tumor necrosis factor alpha (TNF-α) treatment) are not effective in all patients. Thus, the need for new therapeutic targets in RA is still high. There is increasing evidence that the non-neuronal cholinergic system (NNCS) might play a role in the pathology of RA. We and others demonstrated that all necessary components for a NNCS such as acetylcholine (ACh) synthesizing and degrading enzymes, choline and ACh transporters as well as different muscarinic and nicotinic ACh receptors, are present in synovial tissue and cartilage of the human joint [2–4]. In different experimental models of arthritis, nicotine was shown to have ameliorating anti-inflammatory effects on arthritis symptoms depending on the mode and time point of application [5–8]. These effects were considered to be associated with stimulation of the alpha 7 nicotinic receptor (α7nAChR) [6]. However, the effect of α7nAChR on arthritis is controversially discussed, as opposite outcomes were published for experimentally induced arthritis in α7nAChR-deficient mice [9, 10] or for application of α7nAChR agonists in arthritis [6, 11]. The role of muscarinic receptors in arthritis has not been analyzed to date.

Muscarinic receptors (MRs) belong to the family of G protein-coupled receptors and can be classified in five distinct subtypes M1R to M5R. M1R, M3R, and M5R were shown to couple to G proteins of the G_q/11_ family, while M2R and M4R preferentially signal through the G_i/0_ proteins [12]. Stimulation of muscarinic receptors was shown to mediate many important physiological functions such as smooth muscle contraction and glandular secretion. Targeting of MRs is a promising treatment strategy for different pathologies such as overactive bladder, chronic obstructive pulmonary disease and Sjögren’s syndrome [13]. Further, muscarinic receptors are expressed on various immune cells [14, 15], and activation of MRs is considered to have mainly pro-inflammatory function [16]. Stimulation of M3R was shown to induce the expression of pro-inflammatory cytokines and chemokines and to subsequently influence the recruitment of neutrophils [17–19]. Interestingly, an upregulation of muscarinic receptor expression was observed on lymphocytes of patients with RA [20]. Further, activation of M3R was reported to induce the proliferation of bronchial fibroblasts [21]. As synovial fibroblasts proliferate strongly during pannus formation in RA, we sought to analyze whether M3R deficiency could be beneficial for arthritis, leading to reduced inflammation and joint destruction.

In the present study we used the model of collagen antibody-induced arthritis (CAIA) to investigate the effect of M3R on arthritis development. CAIA is a fast and highly synchronic model to study the inflammatory phase of arthritis also in mice with C57BL/6 background, which are usually less responsive for induction of experimental arthritis [22]. While clinical signs of arthritis were not significantly different between the two genotypes, arthritic M3R-deficient mice showed stronger joint destructive effects and a tendency toward a higher inflammatory response compared to wild-type (WT) mice with CAIA.

## Methods

### Animals

Mice deficient for M3R (B6.129S6-CF1-Chrm3^tm1Jwe^; M3R^−/−^) and WT littermates (C57BL/6N_Tac_; WT) were obtained from Prof. Dr. J. Wess (National Institutes of Health, Bethesda, MD, USA). Mice were housed under pathogen-free conditions, a 12 h light–dark cycle and received food and water ad libitum. All experiments were approved by the local ethics committee (Regierungspräsidium Giessen, GI20/28 Nr. 52–2013).

### Collagen antibody-induced arthritis (CAIA)

CAIA was induced in 12-week-old M3R^−/−^ and WT mice (n = 10; 6 males and 4 females for each genotype) by an intraperitoneal (i.p.) injection of 0.2 mg/g body weight anti-type II collagen 5-clone monoclonal antibody cocktail (Chondex, Redmond, WA, USA) on day 0. Control mice (n = 7; 5 males and 2 females for each genotype) received phosphate-buffered saline (PBS) instead of the anti-collagen antibody cocktail. On day 3 all mice recieved an i.p. injection of 2 µg/g body weight lipopolysaccharide (LPS; *E.coli *0111:B4M; Chondrex). The animals were scored every day for manifestation of arthritic symptoms in the paws as published by Irmler et al. [23]. Briefly, for each paw the swelling in wrist/ankle joints and in the metacarpophalangeal or metatarsophalangeal joints were graded independently with a score from 0 to 3 (0 = no redness or swelling; 1 = slight swelling and redness; 2 = strong swelling and redness; 3 = massive swelling and redness). The number of affected digits or toes was divided by half and added to the score of each paw. The mice were further scored on a daily basis according to their overall condition. The total condition score included the assessment of body weight, general condition, spontaneous behavior and arthritis score (total condition score of 0–4 = no handicap; 5–9 = low handicap; 10–19 moderate handicap; ≥ 20 strong handicap). On day 10 the animals were killed and samples were taken for analysis. Mice were considered to be arthritic when they reached a cumulative arthritis score (CAS) ≥ 4. As the number of female animals that reached the critical cumulative arthritis score was very low, only male mice were used for further investigation. Paws from arthritic animals were taken for analyses when they reached a single paw arthritis score (SPAS) ≥ 2.

### Real-time reverse transcription polymerase chain reaction (RT-PCR)

Total RNA was isolated from the paws using the RNA Lipid Tissue Mini Kit (Qiagen, Hilden, Germany). The Quantitect kit (Qiagen) was used for removal of DNA contaminations and subsequent reverse transcription according to the manufacturer’s protocol. Real-time RT-PCR analysis was performed using the QuantiFast PCR Kit (Qiagen) and the I-Cycler IQ5™ detection system (Bio-Rad, Munich, Germany). The following mouse gene-specific primers were used for amplification: *Il6* (152 base pairs (bp); NM_031168.1) forward 5′-CCTCTCTGCAAGAGACTTCCATCGA-3′, reverse 5′-AGCCTCCGACTTGTGAAGTGGT-3′; *Cxcl2* (146 bp; NM_009140.2) forward 5′-GCGCCCAGACAGAAGTCATAGCC-3′, reverse 5′-CAGCAGCCCAGGCTCCTCCT-3′; *Rankl* (86 bp; NM_011613.3) forward 5′-AAGCCTTTCAGGGGGCCGTG-3′, reverse 5′-GCCTTCCATCATAGCTGGAGCTCCT-3′; *CtsK* (81 bp; NM_007802.3) forward 5′-CAGAGTGGGAAGGCAGGGTCCC-3′, reverse 5′-ACTGGCCCTGGTTCTTGACTGGA-3′; *Mmp13* (125 bp; NM_008607.2) forward 5′-AGGACCCAGGAGCCCTGATGTT-3′, reverse 5′-AGGGTTGGGGTCTTCATCGCCTG, *β-actin* (165 bp; NM_007393.3) forward 5′-TGTTACCAACTGGGACGACA-3′, reverse 5′-GGGGTGTTGAAGGTCTCAAA-3′.

Standard and melt curves were performed to determine PCR efficiency and specificity of amplification, respectively. Mean cycle thresholds (CT) values were normalized to the reference gene *β-actin* (dCT).

### FACS analysis of leukocytes in blood

Blood was collected in heparinized tubes and before centrifugation, 10 μl of blood sample were taken for determining the absolute number of leukocytes using CD45-FITC antibody (BioLegend, Fell, Germany) and AccuCount particles (Spherotech Inc., Lake Forest, IL, USA). After centrifugation plasma was taken and the cell pellet was depleted of erythrocytes by two treatment steps with 50 ml erythrocyte lysis buffer (0.15 M NH_4_Cl, 10 mM KHCO_3_, 0.1 mM Na_2_EDTA pH 7.3). After washing with PBS, cells were incubated for 10 min on ice with FACS buffer (1 % fetal bovine serum in PBS) containing 1 μg of purified anti-mouse CD16/CD32 Fc block (eBioscience, Frankfurt am Main, Germany) per 10^6^ cells. Cells were subsequently stained for 30 min at 4 °C in the dark with CD45-APC-Cy7, GR1-BV510 and Ly6C-PerCP-Cy5.5 (all from BioLegend) and then fixed for 10 min at room temperature with 1 % paraformaldehyde (PFA; Merck, Darmstadt, Germany) in PBS. Samples were analyzed using FACS Canto II flow cytometer and FACS Diva software (BD Bioscience, Heidelberg, Germany).

### Enzyme-linked immunosorbent assay (ELISA)

Interleukin-6 (IL-6) concentration in plasma and paw homogenates was measured using mouse-specific ELISA kit (R&D Systems, Wiesbaden, Germany). Paws were lysed in 20 mM Tris/HCl pH 8.0 containing 137 mM NaCl, 5 mM EDTA, 10 % glycerol, 1 % Triton-X 100, 1 mM dithiothreitol and 1 mM PMSF. For tissue ELISA, IL-6 concentration was normalized to total protein content of the paw homogenate, which was determined using the DC protein assay (Bio-Rad) according to the manufacturer’s protocol.

### Acetylcholine measurement

Samples were prepared for acetylcholine measurement as published by Klein et al. [24, 25]. Briefly, paws were homogenized for 5 × 20 s in ice-cold methanol/chloroform (2:1; 3 vol/g wet weight) followed by an addition of 1 vol H_2_0 and chloroform and subsequent homogenization. Hydrophilic phase and lipophilic phase were separated by centrifugation [25]. The upper hydrophilic phase was dried by vacuum centrifugation, dissolved in HPLC buffer (50 mM KHCO_3_, 1.6 mM sodium decanesulfonate and 0.17 mM EDTA pH 8.3) and then subjected to HPLC measurement using an Eicom HTEC-500 microbore system coupled to a Shimadzu SIL-20 AC autosampler [26]. The detection of the system was 1–2 fmol.

### Histological analysis

Knee joints were fixed in 4 % phosphate-buffered PFA and demineralized in 0.281 M Tris-buffer containing 10 % EDTA (Merck). Sections were cut into a thickness of 3 μm, deparaffinized, rehydrated, and routinely stained with hematoxylin and eosin (H&E), alcian blue/PAS (periodic acid-Schiff), toluidine blue staining or were used for enzyme- or immunohistochemistry. Determination of osteoclasts was performed using enzyme histochemistry for tartrate-resistant acidic phosphatase (TRAP). Sections were pretreated with sodium acetate buffer (pH 5.2) for 10 min and subsequently incubated for 45 min at 37 °C in a solution containing Naphtol AS-TR phosphate (Sigma-Aldrich, Taufkirchen, Germany), N,N-dimethylformamid (Sigma-Aldrich), sodium tartrate (Merck) and Fast Red TR Salt (Sigma-Aldrich). Sections were counterstained with hematoxylin (Shandon Scientific Ltd, Runcorn, UK) and coverslipped with Kaiser’s glycerol gelatin (Merck).

Collagen II, alpha-smooth muscle actin (α-SMA), neutrophils, matrix metalloproteinase 13 (MMP13), cathepsin K and receptor activator of nuclear factor-κB ligand (RANKL) were stained using immunohistochemistry. For RANKL staining slides were subjected to heat-induced antigen retrieval with citric buffer (pH 6.0) for 1 h at 60 °C prior to further treatment. All sections were treated with 3 % H_2_0_2_ to block endogenous peroxidase followed by incubation with 0.2 % hyaluronidase (Biologo, Kronshagen, Germany) for collagen II or incubation with 1 % bovine serum albumin (BSA)/PBS for neutrophil staining. Slides were then subjected to overnight incubation at 4 °C with the following primary antibodies: rabbit anti-α-SMA (1:100; Acris, Hiddenhausen, Germany), polyclonal rabbit anti-collagen II (1:300; Biologo), rat anti-neutrophil (1:100; NIMP-R14, Abcam, Cambridge, MA, USA), rabbit anti-MMP13 (1:50, Abcam), goat anti-cathepsin K (1:250, Santa Cruz Biotechnology, Inc., Heidelberg, Germany) and rabbit anti-RANKL (1:500, GeneTex, Irvine, CA, USA). Further, sections were subjected for 45 min to biotinylated goat anti-rabbit (1:500; Vector Laboratories, Burlingame, CA, USA), biotinylated rabbit anti-rat (1:150; Dako, Hamburg, Germany) or biotinylated rabbit anti-goat antibody (1:800, Dako) and subsequently treated with Vectastain ABC Elite Kit (Vector Laboratories) for 30 min at room temperature according to the manufacturer’s protocol. Staining was visualized using Nova Red (Vector Laboratories), counterstained with hematoxylin and DePex (Serva, Heidelberg, Germany) was used for coverslipping.

Sections were evaluated with a photomicroscope (Axiophot 2; Zeiss, Jena, Germany) equipped with a digital camera (DC500; Leica, Bensheim, Germany). For histopathological evaluation of arthritis severity, sections were scored in a blinded manner regarding changes in the synovial membrane, cartilage degradation, bone erosions and synovial blood vessels (Table 1). For quantification of neutrophils, the number of immunopositive cells was counted per high-power field (magnification: 400×).

**Table 1:** Histological score for evaluation of pathological changes in synovial membrane, cartilage, bone and blood vessels

Score	Synovial membrane	Cartilage	Bone	α-SMA
0	No changes	No changes	No changes	Few blood vessels, none with thickened walls
1	>2 cell layers, moderate immune cell infiltration	Erosion in parts of cartilage surface	Bone remodeling only close to the joint capsule	Few blood vessels, few with thickened walls
2	>5 cell layers, strong immune cell infiltration, starting pannus formation	Erosion of cartilage surface, cartilage destruction	Bone remodeling also in deeper parts reaching epiphyseal plate	Increased number of blood vessels, some of them with thickened walls
3	Like score 2 + pannus formation + more blood vessels	Like score 2 + cartilage covered by connective tissue	Like score 2 + bone degradation	Many blood vessels, most of them (more than 50 %) with thickened walls

*α-SMA* alpha-smooth muscle actin

### Transmission electron microscopy (TEM)

Knee joints were fixed in yellow fix (2 % PFA, 2 % glutaraldehyde and 0.02 % picric acid), washed with 0.1 M cacodylate buffer (pH 7.2) and subsequently fixed with 1 % osmium tetroxide (OsO4; Fluka, Buchs, Switzerland). Specimens were dehydrated and embedded in Epon (Serva, Heidelberg, Germany). Ultra-thin sections (70–80 nm) were cut, contrasted with uranyl acetate and lead citrate (Reichert Ultrastainer, Leica, Germany) and analyzed using a Zeiss EM 912 transmission electron microscope (Zeiss, Oberkochen, Germany) with a digital camera (2 K, TRS; Albert Troendle Prototypentwicklung, Moorenweis, Germany).

### Statistical analysis

For statistical analysis the nonparametric Kruskal–Wallis test was performed followed by the Mann–Whitney *U* test. A *P* value < 0.05 was considered significant. Analyses were performed using GraphPad Prism software (version 6.0; GraphPad Software Inc., La Jolla, CA, USA).

## Results

### Clinical data

**Fig. 1:**
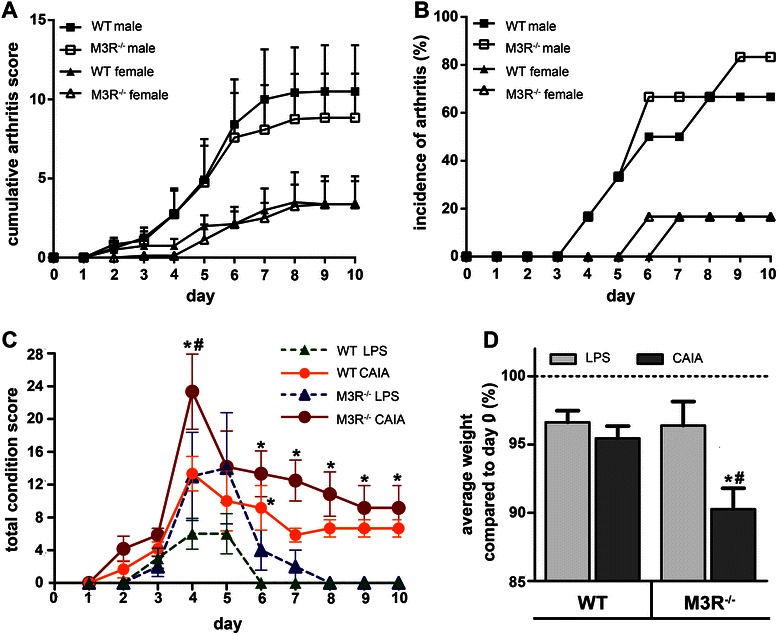
Mice deficient for M3R were not protected from clinical signs of arthritis. **a** Clinical arthritis score for WT and M3R^−/−^ mice with CAIA. Male (n = 6 for each genotype) and female (n = 4 for each genotype) mice of the respective CAIA group were taken for analysis. The cumulative arthritis score (CAS) on each day of experiment is represented as mean ± SEM. **b** Incidence of arthritis development in male (n = 6 for each genotype) and female (n = 4 for each genotype) WT and M3R^−/−^ mice with CAIA. Incidence is given as percentage of mice per group that reached a CAS ≥ 4 on the indicated day of experiment. **c** Total physical condition score of male LPS control (n = 5 for each genotype) and arthritic (CAS ≥ 4) WT (n = 4) and M3R-deficient (n = 5) mice. The total condition score for every day of experiment is given as mean ± SEM. *Asterisks* indicate statistical significance (*P < 0.05: CAIA vs. respective LPS control; ^*#*^ *P* < 0.01: M3R^−/−^-CAIA vs. WT-CAIA). **d** Average body weight of male LPS control and arthritic M3R^−/−^ and WT mice. For each group the average percentage of body weight throughout the experiment compared to experimental day 0 is given as mean ± SEM. *P < 0.05: CAIA vs. respective LPS control; ^*#*^ *P* < 0.05: M3R^−/−^-CAIA vs. WT-CAIA. *CAIA * collagen antibody-induced arthritis, *LPS* lipopolysaccharide, *M3R* M3 muscarinic acetylcholine receptor, *WT* wild-type

CAIA was induced in male and female WT and M3R-deficient mice. In general male mice developed stronger arthritic symptoms (Fig. 1a) and had a higher incidence of arthritis development (cumulative arthritis score ≥ 4) compared to female mice treated the same (Fig. 1b). There was, however, no significant difference in the clinical arthritis score between WT and M3R^−/−^ mice for either sex (Fig. 1a). Interestingly, on day 6 of the experiment, M3R-deficient mice showed a higher incidence of arthritis (male: 66 % for M3R^−/−^ vs. 50 % for WT; female: 20 % for M3R^−/−^ vs. 0 % WT mice) and also at the end of experiment, disease was induced in 80 % of male M3R^−/−^ mice, while only 66 % of male WT mice developed arthritic symptoms (Fig. 1b). As only 20 % of female mice developed arthritic symptoms, we decided to continue the study using only male mice. Generally, the overall physical condition of male M3R^−/−^ mice seemed to be more affected by CAIA induction. Especially on day 4, one day after LPS injection, the total body condition score was significantly enhanced in M3R-deficient compared to WT mice (Fig. 1c). This was further reflected by the weight loss observed for M3R-deficient mice, as over the whole experiment the average body weight of arthritic M3R^−/−^ mice was significantly reduced compared to WT mice with arthritis (Fig. 1d). Acetylcholine was detectable in paws of all animals in a range between 19.2 and 288.3 pmol/g wet weight (97.24 ± 18.66, mean ± SEM, n = 20). However, due to large variations between samples, there were no significant differences between individual groups.

### Histopathological changes in synovial membrane

**Fig. 2:**
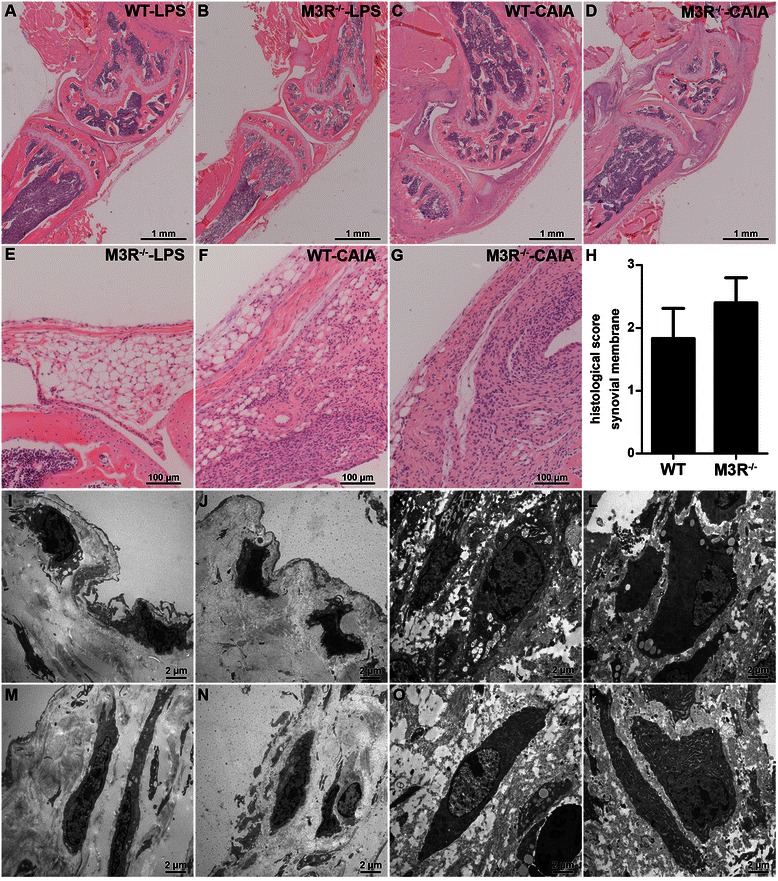
Synovial changes in WT and M3R-deficient mice with CAIA. **a**–**g** Hematoxylin and eosin (H&E) staining of knee joint sections from male WT-LPS (**a**), M3R^−/−^-LPS (**b**, **e**), WT-CAIA (**c**, **f**) and M3R^−/−^-CAIA (**d**, **g**) mice. Original magnification: 25× (**a**–**d**) and 200× (**e**–**g**). **h** Quantification of histopathological changes in knee joint synovial tissue of male arthritic WT and M3R^−/−^ mice. Data are given as mean histopathological score ± SEM. **i**–**p** Transmission electron microscopy of synovial macrophages (**i**–**l**) and fibroblasts (**m**–**n**) of male WT-LPS (**i** and **m**), M3R^−/−^-LPS (**j** and **n**), WT-CAIA (**k** and **o**) and M3R^−/−^-CAIA (**l** and **p**) mice. Original magnification: 5000×. *CAIA* collagen antibody-induced arthritis, *LPS* lipopolysaccharide, *M3R* M3 muscarinic acetylcholine receptor, *WT* wild-type

Histological analysis of knee joints revealed classical synovial changes in arthritic WT and M3R-deficient mice compared to nonarthritic LPS controls (Fig. 2). While there was only a thin layer of synovial cells in LPS control animals, arthritic mice displayed a strong thickening and pannus formation of synovial membrane. Interestingly, the synovial tissue of arthritic M3R-deficent mice seemed to show a tendency toward more severe pathological changes, which were, however, not statistically significant when compared to arthritic WT mice (Fig. 2h). Ultrastructural analysis of synovial macrophages and fibroblasts clearly showed hypertrophy of both cell types in arthritic animals (Fig. 2i–p). In mice with CAIA a higher number of vacuoles and endoplasmatic reticulum was observed in synovial macrophages and fibroblasts, respectively.

**Fig. 3:**
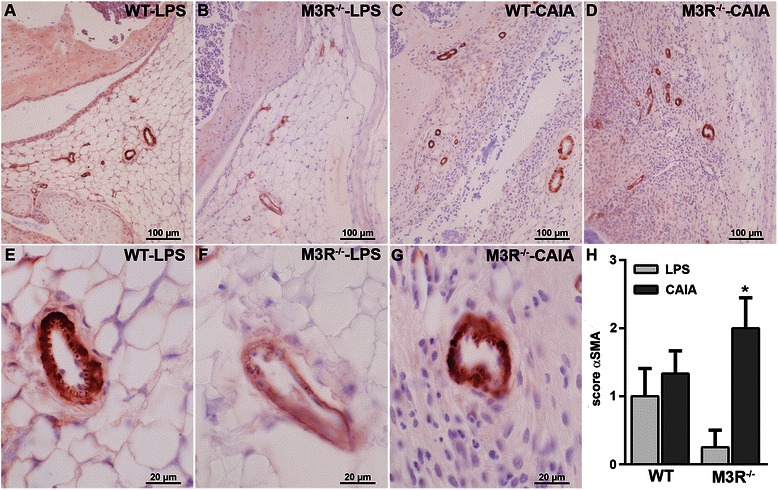
Analysis of α-SMA positive blood vessels in arthritic and nonarthritic WT and M3R^−/−^ mice. **a**–**g** Immunohistochemical staining for α-SMA in knee joints of male WT-LPS (**a**, **e**), M3R^−/−^-LPS (**b**, **f**), WT-CAIA (**c**) and M3R^−/−^-CAIA (**d**, **g**) mice. Original magnification: 200× (**a**–**d**) and 1000× (**e**–**g**). **h** Histopathological score for α-SMA staining. Data are given as mean histopathological score ± SEM. *P < 0.05 CAIA vs. respective LPS control. *CAIA* collagen antibody-induced arthritis, *LPS* lipopolysaccharide, *M3R* M3 muscarinic acetylcholine receptor, *WT* wild-type, *α-SMA* alpha-smooth muscle actin

One characteristic feature of arthritis is an increase in the amount of blood vessels in the diseased synovial tissue. Thus, we performed immunohistochemistry for α-SMA, labeling vascular smooth muscle cells (Fig. 3). The staining and respective histopathological scoring (Fig. 3h) revealed a significantly enhanced number of α-SMA-positive blood vessels including vessels with thickened walls in M3R^−/−^-CAIA mice when compared to respective LPS control mice. Interestingly, compared to M3R^−/−^-LPS mice, WT-LPS control mice showed a higher histopathological score, which was not markedly enhanced by CAIA induction (Fig. 3e and h).

### Inflammatory response

**Fig. 4:**
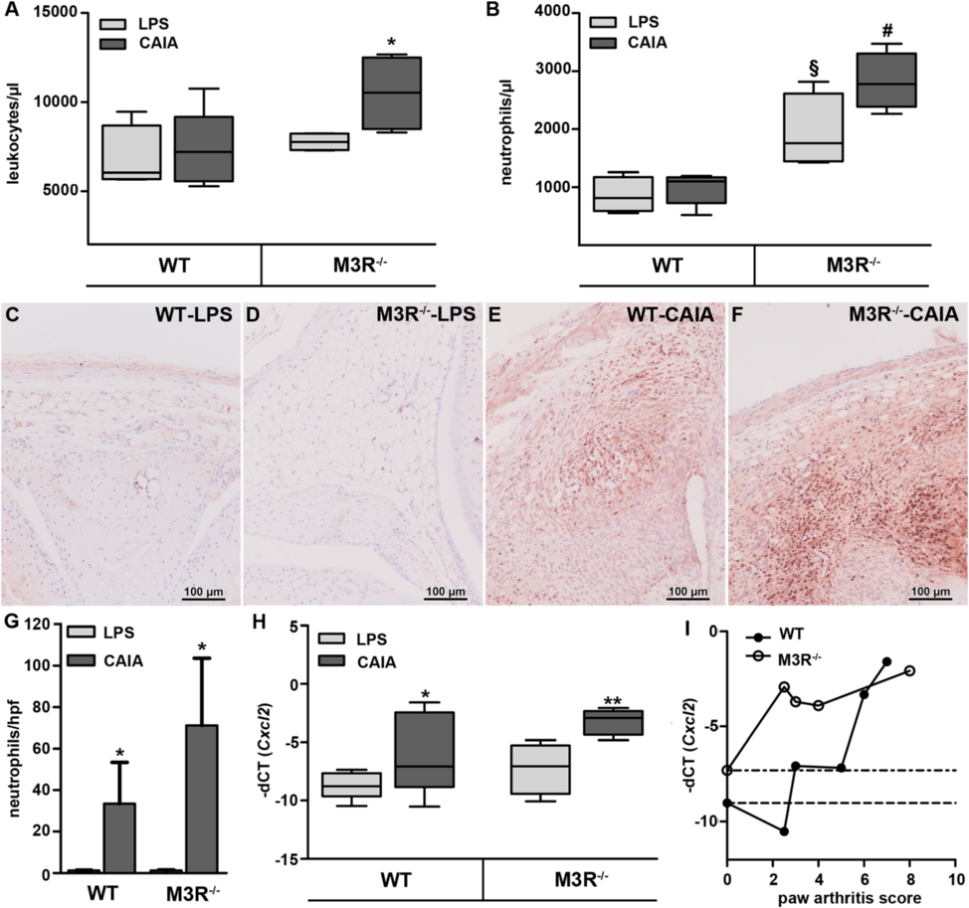
Higher number of pro-inflammatory cells in circulation and joints of M3R-deficient mice. **a**, **b** Quantification of FACS analysis of total CD45+ (**a**) and CD45 + Ly6C + GR1+ (**b**) cells in whole blood from male LPS control and arthritic M3R^−/−^ and WT animals. Data are given as box plots with the median indicated by a *solid line* within the box. *P < 0.05 CAIA vs. respective LPS control; ^*#*^ *P* < 0.05: M3R^−/−^-CAIA vs. WT-CAIA; ^§^ *P* < 0.05: M3R^−/−^-LPS vs. WT-LPS. **c**–**f** Immunohistochemical staining for neutrophils in knee joints of male WT-LPS (**c**), M3R^−/−^-LPS (**d**), WT-CAIA (**e**), M3R^−/−^-CAIA (**f**) mice. Original magnification: 200×. **g** Quantification of neutrophil staining with data given as average number of positive cells per high-power field (hpf) ± SEM. *P < 0.05 CAIA vs. respective LPS control. **h** Real-time RT-PCR analysis of *Cxcl2* mRNA expression in paws of male WT and M3R^−/−^ mice with CAIA and respective LPS-treated control mice, normalized to *β-actin* mRNA. Data are given as –dCT values presented as box plots. *P < 0.05 and **P < 0.01 CAIA vs. respective LPS control. **i** Correlation of *Cxcl2* mRNA expression with the arthritis score of the respective paw in arthritic WT and M3R^−/−^ mice. *Dotted lines* indicate the mean *Cxcl2* mRNA expression of the corresponding LPS-treated control mice. *CAIA* collagen antibody-induced arthritis, *LPS* lipopolysaccharide, *M3R* M3 muscarinic acetylcholine receptor, *WT* wild-type

Induction of arthritis led to a substantial increase of total leukocyte number in the blood of M3R-deficient, but not of WT mice (Fig. 4a). Further, the number of circulating neutrophils was not significantly changed by arthritis induction in WT mice (Fig. 4b). Interestingly, in the blood of M3R-deficient LPS control mice, neutrophil count was markedly increased compared to WT mice. Induction of arthritis led to a further, modest, but not statistically significant enhancement of circulating neutrophils in M3R-deficient mice (Fig. 4b). Immunohistochemistry for neutrophils demonstrated that arthritis induction resulted in a strong infiltration of neutrophils in the knee joint of arthritic animals (Fig. 4c–g). Quantification of immunopositive cells revealed higher numbers of neutrophils in knee joints of arthritic M3R-deficient mice when compared to WT-CAIA mice. However, this difference was not statistically significant, due to a large variation between samples (Fig. 4g). We further found that mRNA expression of chemokine (C-X-C motif) ligand 2 (CXCL2), a main chemoattractant for neutrophils, was strongly upregulated in paws of arthritic mice (Fig. 4h). Correlating the expression of *Cxcl2* with the arthritis scores of the respective paws revealed that *Cxcl2* expression in paws of arthritic M3R-deficient mice was already markedly enhanced in paws with low arthritis score (Fig. 4i). In WT mice, however, strong induction of *Cxcl2* expression was only observed in paws with higher arthritis score (Fig. 4i).

**Fig. 5:**
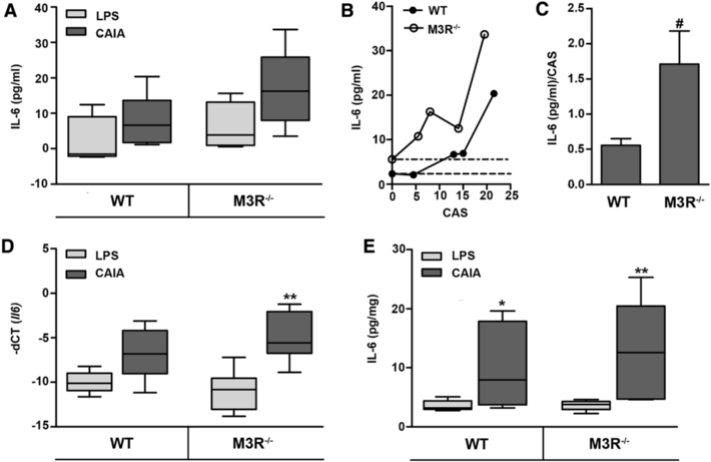
Effect of M3R-deficiency on pro-inflammatory cytokine IL-6 in mice with CAIA. **a** Enzyme-linked immunosorbent assay (ELISA) measurement of IL-6 concentration in plasma of male arthritic WT and M3R^−/−^ mice and respective LPS-treated control mice. **b** Correlation of circulating IL-6 abundance with the respective cumulative arthritis score (CAS) of arthritic WT and M3R^−/−^ mice. *Dotted lines* indicate the mean IL-6 concentration of the respective LPS-treated control mice. **c** Ratio of plasma IL-6 level and CAS in arthritic male WT and M3R^−/−^ mice given as mean ± SEM. ^*#*^ *P* < 0.05: M3R^−/−^-CAIA vs. WT-CAIA. **d** Real-time reverse transcription polymerase chain reaction (RT-PCR) analysis of *Il6* mRNA expression in paws of male WT and M3R^−/−^ mice with CAIA and respective LPS-treated control mice, normalized to *β-actin* mRNA. **P < 0.01 CAIA vs. respective LPS control. **e** ELISA measurement of IL-6 concentration in paw homogenates of male LPS-treated and arthritic WT and M3R^−/−^ mice. Data were normalized to total protein concentration. *P < 0.05 and **P < 0.01 CAIA vs. respective LPS control. *CAIA * collagen antibody-induced arthritis, *CAS* cumulative arthritis score, *IL-6* interleukin-6, *LPS* lipopolysaccharide, *M3R* M3 muscarinic acetylcholine receptor, *WT* wild-type

ELISA measurement of circulating pro-inflammatory cytokine interleukin (IL)-6 revealed a modest, but not statistically significant, increase in plasma of arthritic mice (Fig. 5a). Interestingly, in mice deficient for M3R, abundance of circulating IL-6 was strongly enhanced in mice with low cumulative arthritis score (CAS), while WT mice only showed high plasma IL-6 levels when they reached a high CAS (Fig. 5b). Thus, the ratio of plasma IL-6 concentration to CAS was significantly higher in M3R-deficient mice compared to WT mice (Fig. 5c). Further, IL-6 mRNA expression was markedly increased in paws of arthritic animals, however, only in paws of M3R-deficient animals was the increase statistically significant (Fig. 5d). ELISA measurements in paw homogenates showed that IL-6 protein was significantly increased in arthritic paws of both WT and M3R-deficient mice, with a tendency toward stronger induction in M3R^−/−^-CAIA mice (Fig. 5e).

### Joint destruction

**Fig. 6:**
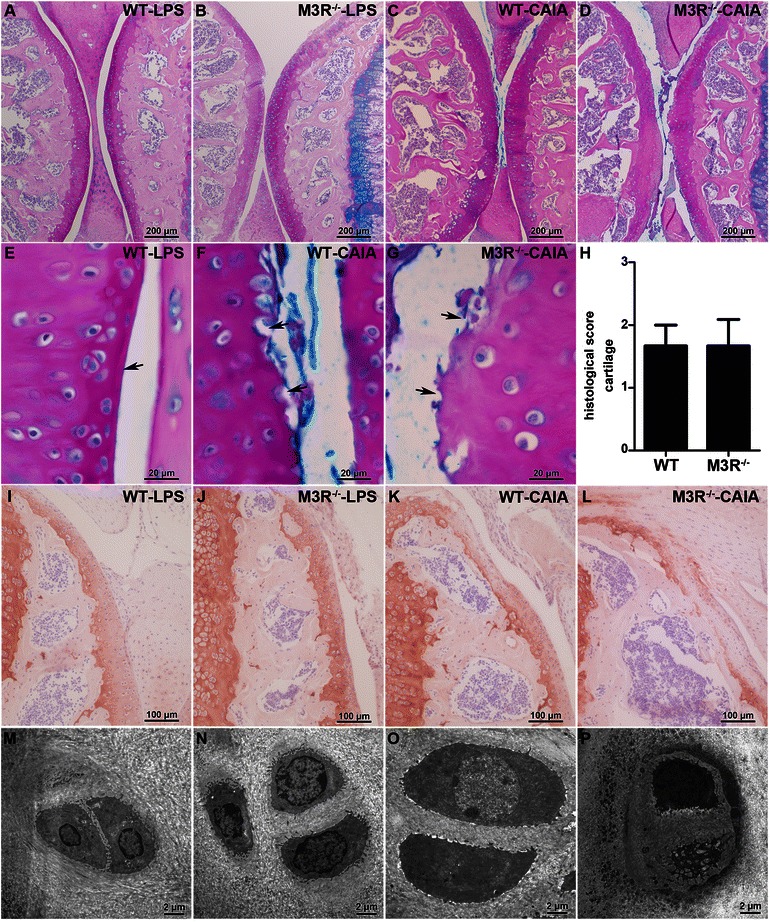
Analysis of cartilage destruction in arthritic WT and M3R^−/−^ mice. **a**–**g** Alcian blue/periodic acid-Schiff (PAS) staining on knee joint sections from male WT-LPS (**a**, **e**), M3R^−/−^-LPS (**b**), WT-CAIA (**c**, **f**), and M3R^−/−^-CAIA (**d**, **g**) mice. Original magnification: 100× (**a**–**d**) and 1000× (**e**–**g**). *Arrows* indicate regions of interest. **h** Histopathological quantification of cartilage destruction in knee joints of male arthritic WT and M3R^−/−^ mice. Data are given as mean of histopathological score ± SEM. **i**–**l** Immunohistochemical staining for collagen II (original magnification 200×) in sections from knee joints of male WT-LPS (**i**), M3R^−/−^-LPS (**j**), WT-CAIA (**k**) and M3R^−/−^-CAIA (**l**) mice. **m**–**p** Transmission electron microscopy of chondrocytes in knee joints of male WT-LPS (**m**), M3R^−/−^-LPS (**n**), WT-CAIA (**o**) and M3R^−/−^-CAIA (**p**) mice. Original magnification: 5000×. *CAIA* collagen antibody-induced arthritis, *LPS* lipopolysaccharide, *M3R* M3 muscarinic acetylcholine receptor, *WT* wild-type

Articular cartilage in knee joints of nonarthritic LPS control animals had a smooth surface (Fig. 6a–b and e), while in arthritic animals the surface was rough and destroyed at many places (Fig. 6c–d and f–g). There was, however, no obvious difference in cartilage destruction between arthritic WT and M3R-deficient mice. We further stained the knee joint sections for collagen II, the main constituent of articular cartilage. Full staining of cartilage was observed in both LPS control groups (Fig. 6i–j) while in arthritic mice parts of cartilage did not show immunoreactivity for collagen II (Fig. 6k–l). This loss of collagen II seemed to be enhanced in M3R^−/−^-CAIA mice when compared to WT-CAIA mice. Taking a closer look at the ultrastructure of chondrocytes (Fig. 6m–p) revealed that, compared to cells of LPS controls and WT-CAIA mice, chondrocytes of arthritic M3R-deficient mice looked less vital, with dense chromatin and cytoplasmic vacuoles (Fig. 6p).

**Fig. 7:**
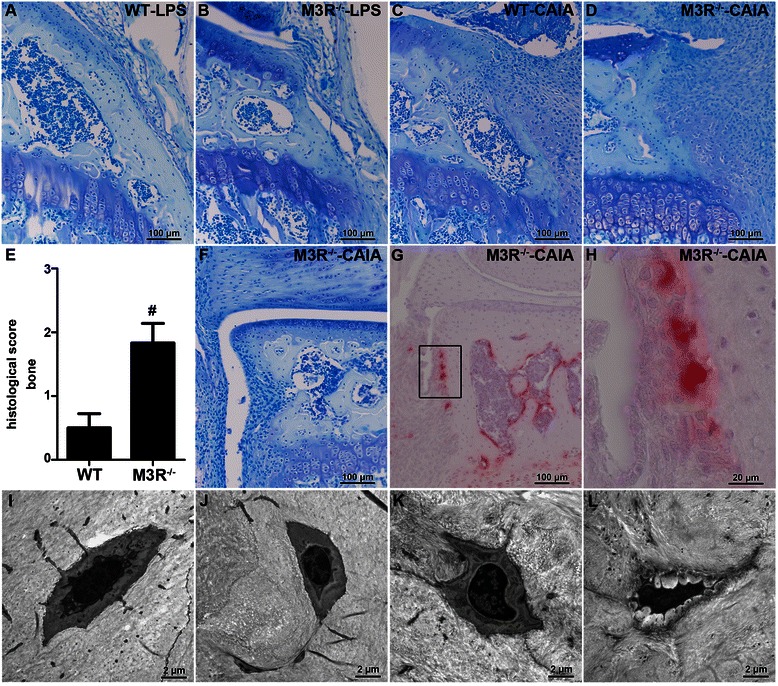
Stronger effect of CAIA on bone destruction in M3R-deficient mice. **a**–**d** Toluidine blue staining on sections from knee joints of male WT-LPS (**a**), M3R^−/−^-LPS (**b**), WT-CAIA (**c**), and M3R^−/−^-CAIA (**d**) mice. Original magnification: 200×. **e** Histopathological quantification of bone destruction in knee joints of male arthritic WT and M3R^−/−^ mice. Data are given as mean of histopathological score ± SEM. ^*#*^ *P* < 0.05: M3R^−/−^-CAIA vs. WT-CAIA. **f**–**h** Toluidine blue staining (**f**) and immunohistochemistry for tartrate-resistant acidic phosphatase (TRAP) (**g** and **h**) on section from knee joint of representative male M3R^−/−^-CAIA mouse. Original magnification: 200× (**f**, **g**) and 1000× (**h**) The *square* in **g** indicates the area shown in **h**. **i**–**l** Transmission electron microscopy of osteocytes in knee joints of male WT-LPS (**i**), M3R^−/−^-LPS (**j**), WT-CAIA (**k**) and M3R^−/−^-CAIA (**l**) mice. Original magnification: 6300×. *CAIA* collagen antibody-induced arthritis, *LPS* lipopolysaccharide, *M3R* M3 muscarinic acetylcholine receptor, *WT* wild-type

Further, toluidine blue-stained sections of knee joints were evaluated regarding the degree of bone destruction (Fig. 7a–d). Remarkably, histopathological quantification of arthritis-induced changes in bone structure revealed that bone erosion was significantly enhanced in arthritic M3R-deficient mice compared to WT mice with CAIA (Fig. 7e). The process of bone degradation seemed to involve osteoclasts, as TRAP-positive multinucleated cells were detected in areas of erosive bone (Fig. 7f–h). While osteocytes of both WT and M3R^−/−^ nonarthritic LPS control animals had normal ultrastructure (Fig. 7i and j), transmission electron microscopy showed that osteocytes in arthritic WT animals had a slightly swollen appearance with little pericellular space around the cytoplasm (Fig. 7k). The same was observed for osteocytes of arthritic M3R-deficient mice, however, here mainly osteocytes with shrinkage of cytoplasm and expanded pericellular space were observed in M3R^−/−^-CAIA mice (Fig. 7l). These osteocytes showed many cytoplasmatic processes, the pericellular space appeared brighter and cells looked generally less vital.

**Fig. 8:**
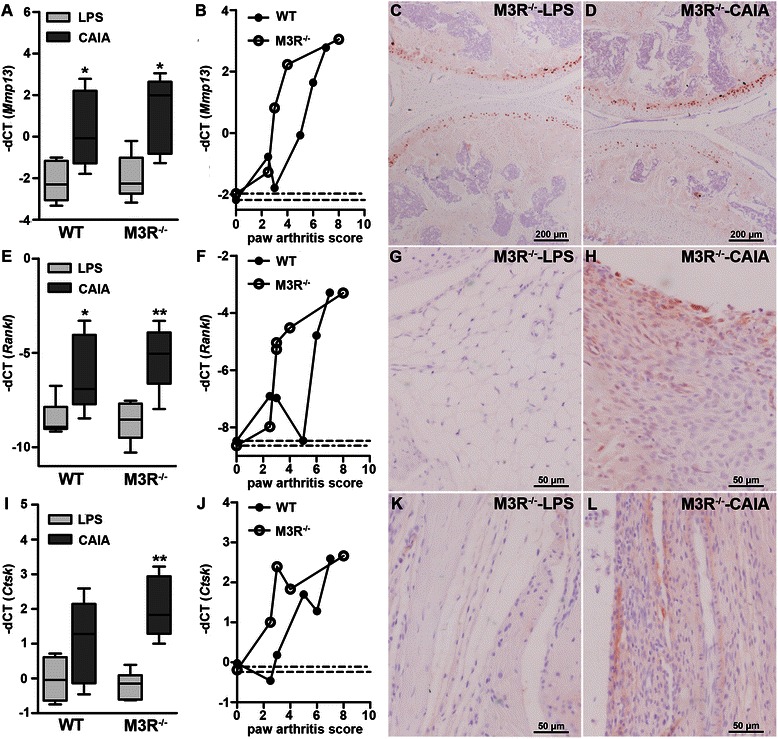
Analysis of markers for joint destruction in arthritic WT and M3R^−/−^ mice. Real-time reverse transcription polymerase chain reaction (RT-PCR) analysis of *Mmp13* (**a**), *Rankl* (**e**), and *Ctsk* (**i**) mRNA expression in paws of male WT and M3R^−/−^ mice with CAIA and respective LPS-treated control mice, normalized to *β- actin* mRNA. *P < 0.05 and **P < 0.01 CAIA vs. respective LPS control. Correlation of *Mmp13* (**b**), *Rankl* (**f**) and *Ctsk* (**j**) mRNA expression with the arthritis score of the respective paw in male arthritic WT and M3R^−/−^ mice. *Dotted lines* indicate the mean mRNA expression of the respective LPS-treated control mice. Immunohistochemical staining for matrix metalloproteinase 13 (MMP13) (**c**–**d**), receptor activator of nuclear factor-κB ligand (RANKL) (**g**–**h**) and cathepsin K (**k**–**l**) in knee joint sections from male M3R^−/−^-LPS (**c**, **g** and **k**) and M3R^−/−^-CAIA (**d**, **h** and **l**) mice. Original magnification: 100× (**c**–**d**) and 400× (**g**–**h** and **k**–**l**). *CAIA* collagen antibody-induced arthritis, * Ctsk* cathepsin K, *LPS* lipopolysaccharide, *M3R* M3 muscarinic acetylcholine receptor, *Mmp13* matrix metalloproteinase 13, *Rankl receptor activator of nuclear factor-κB ligand*, *WT* wild-type

Expression of genes involved in bone destruction, namely matrix metalloproteinase 13 (MMP13), receptor activator of nuclear factor-κB ligand (RANKL) and cathepsin K (CtsK) was further analyzed in paws of control and arthritic M3R^−/−^ and WT animals by real-time RT-PCR and immunohistochemistry (Fig. 8). *Mmp13* mRNA expression in paws was markedly enhanced by arthritis induction in both WT and M3R-deficient mice. Interestingly, in M3R-deficient mice, mRNA expression of *Mmp13* was already prominently induced in paws with low arthritis scores while in WT animals only paws with an arthritis score > 4 showed high levels of *Mmp13* (Fig. 8b). Immunohistochemical staining in the knee joints showed that MMP13 protein was localized mainly in the chondrocytes of the articular cartilage. However, using this method, we were not able to find clear differences between LPS control and arthritic mice (exemplarily shown for M3R-deficient mice in Fig. 8c–d). *Rankl* mRNA expression was significantly enhanced by arthritis induction in both WT and M3R-deficient mice (Fig. 8e), but same as for *Mmp13* mRNA, M3R-deficient mice reached higher expression levels of *Rankl* already in paws with low arthritis score (Fig. 8f). Immunohistochemistry for RANKL protein revealed its localization primarily in synoviocytes (Fig. 8g–h) and chondrocytes of the knee joint, showing a higher abundance in arthritic mice when compared to LPS control mice, confirming the mRNA data. *Ctsk* mRNA expression was significantly induced only in arthritic M3R-deficient mice (Fig. 8i) and also here, the expression was comparably high already in paws with low arthritis scores (Fig. 8j). Cathepsin K protein was localized immunohistochemically in synoviocytes (Fig. 8k–l) as well as chondrocytes and showed a higher abundance in arthritic mice when compared to LPS control mice.

## Discussion

In the present study we first found that M3R-deficient mice responded to collagen antibody-induced arthritis similarly to WT mice. Upon further characterization, however, M3R^−/−^ mice displayed a tendency toward a higher inflammatory response than WT mice. Furthermore, arthritis-induced joint destruction was significantly stronger in mice with M3R deficiency.

No difference in clinical arthritis scores was observed between WT and M3R-deficient mice, but M3R deficiency led to a slightly higher incidence of disease induction. According to previous studies, no sex differences occur in C57BL/6 mice when using LPS boost injection to induce arthritis [27, 28]. When CAIA was induced in both male and female mice of each genotype, however, we clearly observed differences in arthritis development between male and female mice. Contrary to the human situation where incidence of RA is higher in female patients, male mice were affected more strongly by CAIA induction than female mice. Such sex-specific effect is known, e.g., for the model of collagen-induced arthritis [29]. Even though the incidence of arthritis was slightly increased in M3R-deficient mice, there were no obvious differences in clinical arthritis score compared to WT mice. As only a small number of female mice developed arthritic symptoms, we further conducted this study using only male mice. The overall physical condition of M3R-deficient mice was more affected by disease induction. Moreover, M3R^−/−^ mice with CAIA showed a significantly stronger reduction in body weight over the whole experiment than WT mice. In general, mice deficient for M3R are known to have a hypophagic and lean phenotype, due to a lack of the central cholinergic effect on food intake and an increase in energy expenditure [30, 31]. This phenotype might be more pronounced when mice are challenged.

Cholinergic control of inflammation is a widely discussed topic and, especially since the “cholinergic anti-inflammatory pathway” was identified by the group of Tracey and coworkers [32–34], the anti-inflammatory role of the α7nAChR was extensively analyzed. Less research was focused on the role of muscarinic receptors, which were reported to have a pro-inflammatory function [16]. In the present study, however, M3R-deficient mice showed a stronger inflammatory response after arthritis induction than WT mice. Expression of the pro-inflammatory cytokine IL-6 and the number of infiltrating neutrophils was markedly increased in arthritic joints of both genotypes, but infiltration of neutrophils was slightly stronger in M3R-deficient mice. Neutrophils are key players in the pathology of CAIA, and depletion of these cells was shown to have a strong beneficial effect [28]. In accordance with the profound neutrophil infiltration in the arthritic joint, we could further demonstrate that mRNA expression of CXCL2, a main chemoattractant for neutrophils, was significantly induced in whole joints of arthritic WT and M3R-deficient mice. Interestingly, in M3R-deficient mice *Cxcl2* expression was already markedly enhanced in paws with low arthritis score, while in WT mice only strongly arthritic paws revealed high expression of this chemokine.

This stronger inflammatory response in mice lacking M3R was surprising, as opposite findings were reported for, e.g., chronic obstructive pulmonary disease (COPD) or LPS-induced endotoxemia, where muscarinic receptor antagonists had anti-inflammatory effects [35, 36]. The total number of circulating leukocytes and, more specifically, of circulating neutrophils was significantly enhanced in the blood of arthritic M3R-deficient mice, but not of WT mice. Surprisingly, in our study, LPS-injected M3R-deficient mice had significantly higher numbers of circulating neutrophils than LPS-treated WT mice. This finding indicates that LPS application without induction of arthritis had a stronger systemic pro-inflammatory effect when M3R was absent. For WT animals a significant increase in immune cells was only observed directly in the affected joint, but not systemically in the blood. The dose of antibody and LPS used to induce arthritis may not have been strong enough to promote a systemic response in WT mice. Alternatively, the systemic inflammatory response may already have been reduced to non-detectable levels at day 10 of experiment, whereas in M3R-deficient mice it remained elevated. The same might be true for the pro-inflammatory cytokine IL-6, which was not significantly elevated in the circulation of arthritic mice at day 10. However, M3R^−/−^-CAIA mice again displayed higher levels of IL-6 than WT mice. One explanation for the observed pro-inflammatory effects in M3R^−/−^ mice could be the lack of central cholinergic activation of M3 receptors as anti-inflammatory effects were reported for centrally acting muscarinic agonists [37, 38].

Synovial tissue showed typical pathology in all arthritic mice, with a tendency toward more pronounced pathological changes in M3R^−/−^-CAIA mice. Pannus-forming synovial tissue is highly vascularized and thickening of blood vessel walls can be observed under arthritic conditions [39]. Many components of the NNCS including the acetylcholine-synthesizing enzyme choline acetyltransferase (ChAT) were reported to be present in the vasculature [40, 41]. Furthermore, nicotine acts as a pro-angiogenic, mainly via α7nAChR, and may be involved in the pathology of atherosclerosis [42, 43]. Activation of M3R in the vasculature can have different functions, depending on the cell type. While M3R on endothelial cells causes vasodilatation, M3R on vascular smooth muscle cells can cause vasoconstriction [44]. In our study, the number of α-SMA-positive blood vessels was significantly increased by CAIA induction in M3R-deficient mice. Interestingly, the α-SMA histopathological score was strongly enhanced in nonarthritic WT-LPS control mice when compared to M3R^−/−^-LPS mice. Kistemaker and colleagues hypothesized that M3R is involved in smooth muscle mass development, mainly because they found that M3R-deficient mice have lower basal levels of α-SMA in airway arteries and bronchial tissue [45]. This is in accordance with the findings in our study, and this low basal level of α-SMA was strongly induced by CAIA, while for WT mice such induction by arthritis was not observed.

Histopathological scoring of cartilage erosion, mainly regarding surface regularity, did not reveal significant differences between WT and M3R^−/−^ mice with CAIA. Collagen II is the major constituent of articular and hyaline cartilage, but when this cartilage is destroyed, fibrocartilage is formed. Fibrocartilage mainly contains collagen I and is more dense and less elastic than hyaline cartilage. Thus, when specifically collagen II was marked using immunohistochemistry, arthritic M3R-deficient mice seemed to show enhanced loss of collagen II in the articular cartilage, when compared to WT mice with CAIA. Loss of collagen II in rheumatoid arthritis is associated with an increase in matrix-degrading enzymes (e.g., MMP13) [46] and a decrease in collagen II synthesis [47]. In previous work, activation of muscarinic receptors induced collagen synthesis in human lung fibroblasts and M3R-deficient mice displayed reduced collagen deposition in allergen-induced airway remodeling [45, 48]. Thus, the enhanced loss of collagen II in M3R^−/−^ mice could be partly due to an impairment of collagen synthesis when mice are subjected to CAIA induction. Moreover, in arthritic M3R^−/−^ mice, mRNA expression of MMP13 was already strongly enhanced in paws with low arthritis score, when compared to WT mice with CAIA, indicating that collagen II degradation might be stronger in M3R-deficient mice. MMP13 protein was mainly expressed by chondrocytes of the knee joint, however, we could not make out clear differences in MMP13 abundance between LPS control and arthritic animals using immunohistochemistry. However, it could be that the amount of MMP13 protein expressed per cell might be increased, but the number of MMP13-expressing cells per se remains the same under pathological conditions.

MMP13 is not only an important factor for cartilage degradation in arthritis, but additionally plays an essential role for the inflammatory response and bone degradation [49, 50]. Accordingly, the histopathological score evaluating bone degradation was significantly enhanced in arthritic M3R^−/−^ mice compared to WT mice with CAIA. Interestingly, osteocytes of arthritic M3R-deficient animals seemed less vital. Not long ago, osteocytes were considered to be functionless, quiescent cells. However, in recent years it became obvious that these cells have many important functions such as mineralization, formation and resorption of bone as well as sensing of mechanical load [51] and these important pathways might be affected in arthritic M3R-deficient mice.

Generally, bone structure is known to be positively affected by activation of M3R, since M3R-deficient mice were shown to have an osteoporosis-like phenotype [52, 53] and M3R was found to be upregulated in osteoporotic rats [54]. Thus, the stronger bone destruction in M3R^−/−^ mice might not be a surprise. On the other hand, Shi et al. reported that the M3R-mediated effect on bone mass is dependent on the sympathetic tone and the β2 adrenergic receptor [52]. The sympathetic nervous system acts as negative regulator of bone mass accrual via the β2 adrenergic receptor on osteoblasts, and activation of M3R evidently dampens the sympathetic tone, leading to an increase in bone mass [52]. It is known that the sympathetic innervation as well as the β2 adrenergic receptor expression is reduced during the course of arthritis [55–57]. Therefore, the effect of M3R on bone mass via this pathway could be reduced as well and might be less relevant in this setting.

Additionally to MMP13, mRNA expression of cathepsin K and RANKL was already strongly increased in M3R-deficient paws with low arthritis score, while in WT animals enhanced expression was only observed in paws with high arthritis score. Immunohistochemically, we confirmed enhanced protein abundance of both cathepsin K and RANKL in synoviocytes and chondroyctes of the arthritic knee joint. Cathepsin K is a cysteine protease which is expressed predominantly by osteoclasts but also by synoviocytes. It plays an important part in bone as well as cartilage degradation and its expression can be upregulated by pro-inflammatory cytokines [58, 59]. RANKL is a key mediator of osteoclast formation and is expressed by many different cell types including osteocytes [60]. RANKL expressed on synovial fibroblasts seems to be primarily responsible for bone erosions during inflammatory arthritis [61]. However, neutrophils were also identified as potent mediators of bone erosions in RA, as these cells were shown to express RANKL [62, 63]. Thus, the enhancement of joint destruction in arthritic M3R-deficient mice could also be partly due to the stronger inflammatory response shown in these mice.

Transferring these observations to the clinical situation, one might speculate that treatment of patients with M3R agonists might possibly have protective effects for RA. Due to a high similarity between the different receptor subtypes, therapeutic targeting of specific muscarinic receptors is challenging. However, recent advances in the development of subtype-specific allosteric agonists and antagonists may pave the way for a successful targeting of M3R [13].

## Conclusions

In this manuscript, we investigated the role of M3R in experimentally induced arthritis. Arthritic M3R^−/−^ mice showed stronger bone destruction, enhanced collagen II loss in articular cartilage and a tendency toward a stronger inflammatory response, even though clinical arthritis score was not changed as compared to arthritic WT mice. Thus, stimulation of M3R might have protective effects on arthritis.

## Abbreviations

ACh: Acetylcholine
bp: Base pair
CAIA: Collagen antibody-induced arthritis
CAS: Cumulative arthritis score
Ctsk: Cathepsin K
CXCL2: Chemokine (C-X-C motif) ligand 2
CT: Cycle threshold
DMARDS: Disease-modifying antirheumatic drugs
ELISA: Enzyme-linked immunosorbent assay
i.p.: Intraperitoneal
IL-6: Interleukin-6
LPS: Lipopolysaccharide
MMP13: Matrix metalloproteinase 13
MR: Muscarinic acetylcholine receptor
nAChR: Nicotinic acetylcholine receptor
NNCS: Non-neuronal cholinergic system
PBS: Phosphate-buffered saline
RA: Rheumatoid arthritis
RANKL: Receptor activator of nuclear factor-κB ligand
RT-PCR: Reverse transcription polymerase chain reaction
SPAS: Single paw arthritis score
TRAP: Tartrate-resistant acidic phosphatase
WT: Wild-type
α-SMA: Alpha-smooth muscle actin
